# Solitary Neurofibroma of the Maxillary Soft Tissue Causing Bone Destruction: A Rare Presentation

**DOI:** 10.7759/cureus.39423

**Published:** 2023-05-24

**Authors:** Ejaz A Mokhtar, Mandeep Singh, Sharique Equbal, Aabid Majeed

**Affiliations:** 1 Oral and Maxillofacial Surgery, All India Institute of Medical Sciences, Patna, Patna, IND; 2 Oral and Maxillofacial Surgery, Dr. B.R. Ambedkar Institute of Dental Sciences and Hospital, Patna, IND; 3 Oral and Maxillofacial Surgery, Buddha Institute of Dental Sciences, Patna, IND; 4 Dentistry, All India Institute of Medical Sciences, Bhubaneswar, Bhubaneswar, IND

**Keywords:** bone destruction, mri, s-100, solitary, neurofibroma

## Abstract

Neurofibroma is a benign tumor of the peripheral nerve. It usually presents as multiple tumors and as a part of von Recklinghausen’s disease. However, rarely, it can occur in solitary form. The clinical diagnosis of solitary neurofibroma poses a challenge to clinicians as a plethora of benign and malignant lesions of the oral cavity have similar clinical presentations. We present a rare case of solitary neurofibroma of the maxillary soft tissue. A systemic clinical examination was performed to rule out neurofibromatosis. Computed tomography showed displaced teeth and bone destruction. Magnetic resonance imaging showed an ill-defined T2 hyperintense lesion involving the posterior aspect of the tooth-bearing alveolar process of the maxilla. The diagnosis of neurofibroma was confirmed by histological analysis and immunohistochemical studies. The tumor was successfully managed with excision. Three years of follow-up showed no recurrence.

Solitary neurofibroma of the oral cavity is a very rare presentation. Neurofibroma in the maxilla with bone destruction is reported for the first time in the literature. This article will add to the current literature due to the atypical location and presentation of the tumor.

## Introduction

Neural tumors of the oral cavity are uncommon. Neurofibromas usually present as multiple tumors. However, rarely, they can occur in solitary form. The frequency of solitary neurofibromas is approximately 6.5% [1,2]. The distinction between solitary neurofibroma and neurofibromatosis is clinical because histological differentiation between the two is often difficult. Clinically, solitary neurofibromas present as mucosal, non-tender, discrete masses. Solitary neurofibroma, by definition, should be single, and it should not be associated with other manifestations of neurofibromatosis. It can present at any age. The most common sites are the tongue and buccal mucosa. The other sites reported in the literature include the maxillary sinus and soft palate. The posterior mandible is the most frequent intraosseous site [3].

Here, we report a unique case of solitary neurofibroma of the left maxillary soft tissue causing bone destruction in the early period and presenting as a malignant tumor at B.R. Ambedkar Institute of Dental Sciences, Patna.

## Case presentation

**Figure 1:**
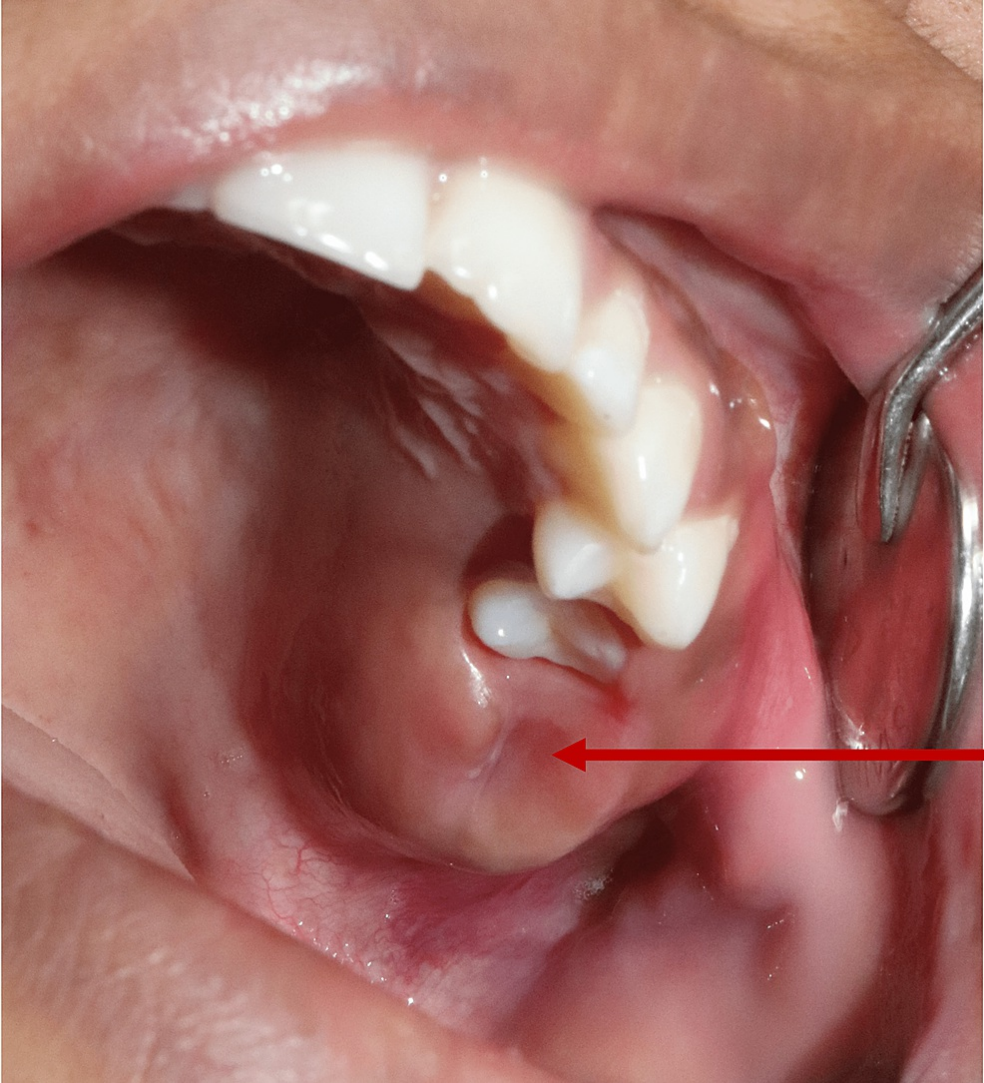
Solitary diffused swelling on the left maxillary alveolar region.

An 18-year-old female was referred from the Ear, Nose, and Throat (ENT) Department at Patna Medical College and Hospital for pain and swelling of the left side of the face for six months. The patient had no previous surgical history or any known disease. Clinical examination showed a swelling of 3 cm × 4 cm in size on the left side of the maxilla posterior to the second premolar tooth (Figure 1).

**Figure 2:**
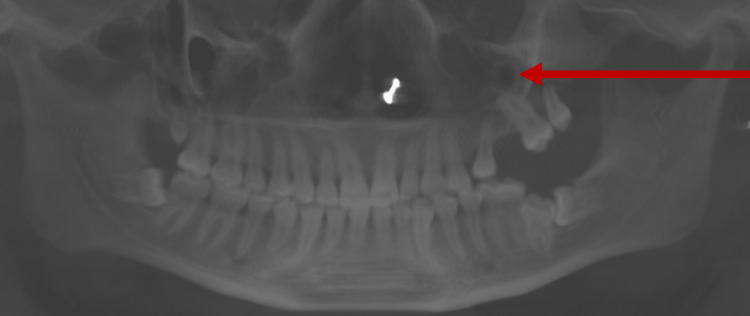
Arrow in panoramic reconstruction generated from cone-beam computed tomography showing tumor displacing the teeth.

The swelling was diffused, tender on palpation, and did not bleed on touch. Further, it was non-compressible and non-fluctuant in nature. The tumor was not associated with any paresthesia. Cone-beam computed tomography (CBCT) showed tumor displacing in the left maxillary alveolar area displacing the teeth (Figure 2).

**Figure 3:**
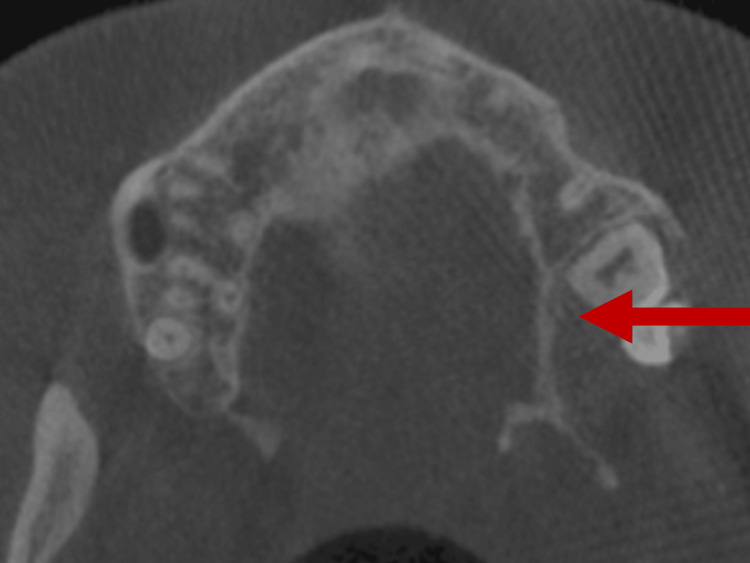
Arrow in cone-beam computed tomography showing the tumor extending into the maxillary sinus and causing bone destruction.

CBCT showed the tumor was extending into the maxillary sinus and causing bone destruction (Figure 3).

**Figure 4:**
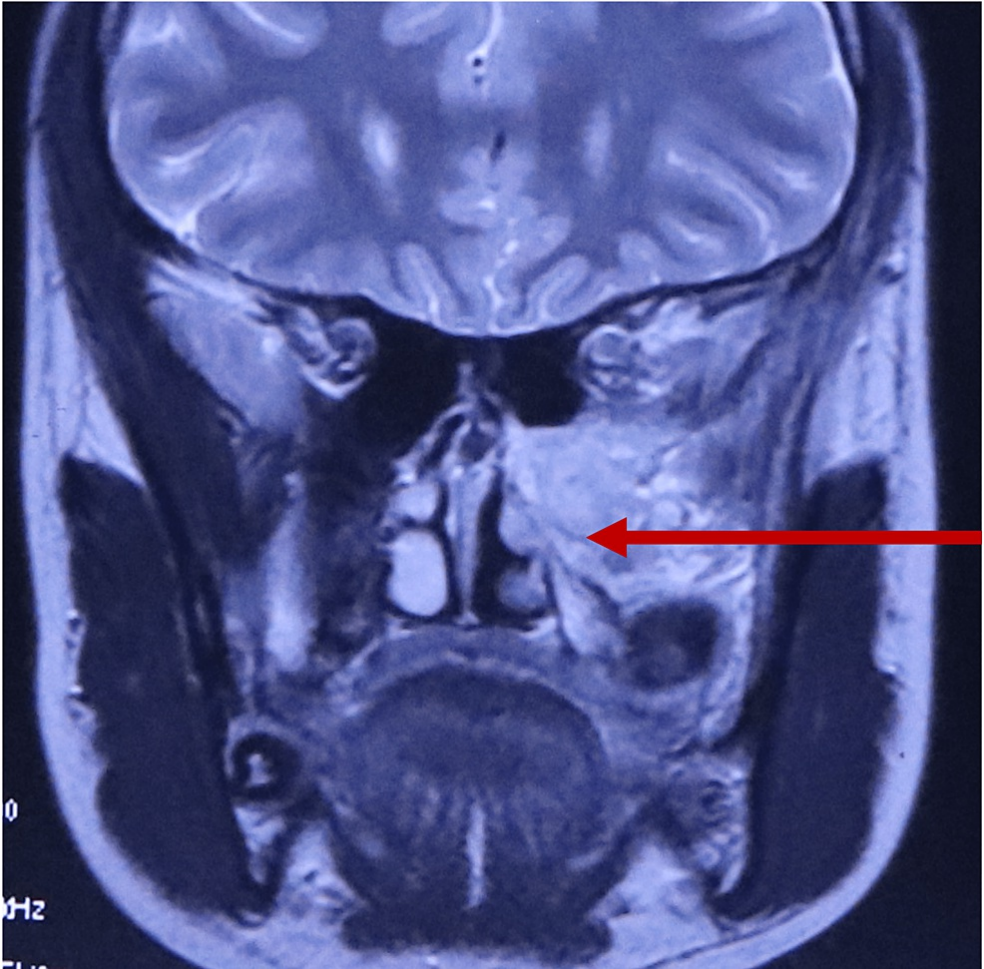
Magnetic resonance imaging showing an ill-defined T2 hyperintense heterogenous lesion involving the posterior aspect of the tooth-bearing alveolar process of the maxilla.

The short history of tumor growth and bone destruction led to a suspicion of malignancy. Magnetic resonance imaging (MRI) showed an ill-defined T2 hyperintense heterogenous lesion involving the posterior aspect of the tooth-bearing alveolar process of the maxilla (Figure 4).

**Figure 5:**
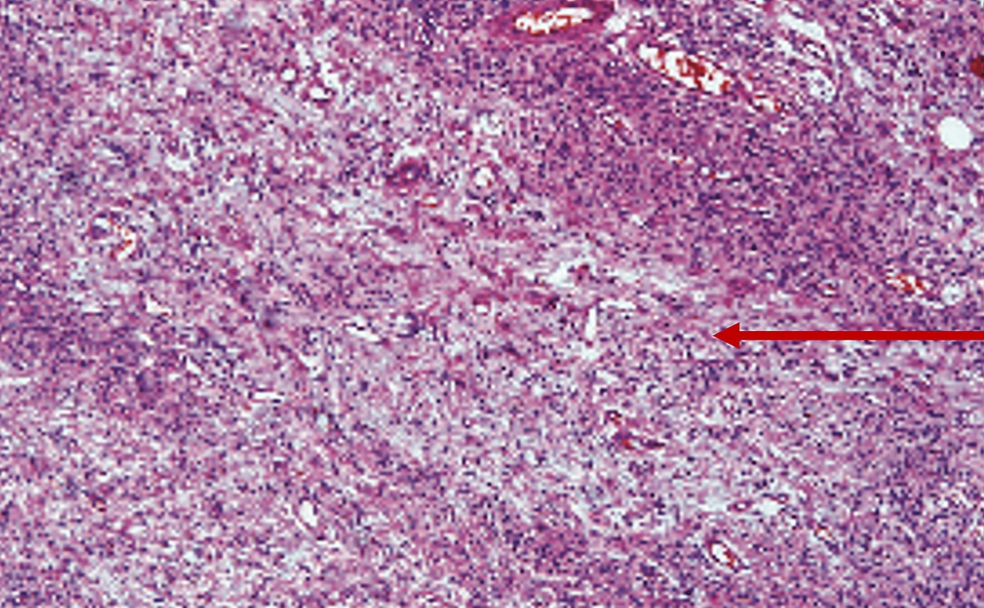
Fibrocellular connective tissue with spindle cells having elongated nuclei.

An incisional biopsy was done. The histopathology yielded spindle cells with wavy hyperchromatic, elongated nuclei and scant cytoplasm, along with fibrocollagenous tissue. Few masts cells were also seen (Figure 5).

**Figure 6:**
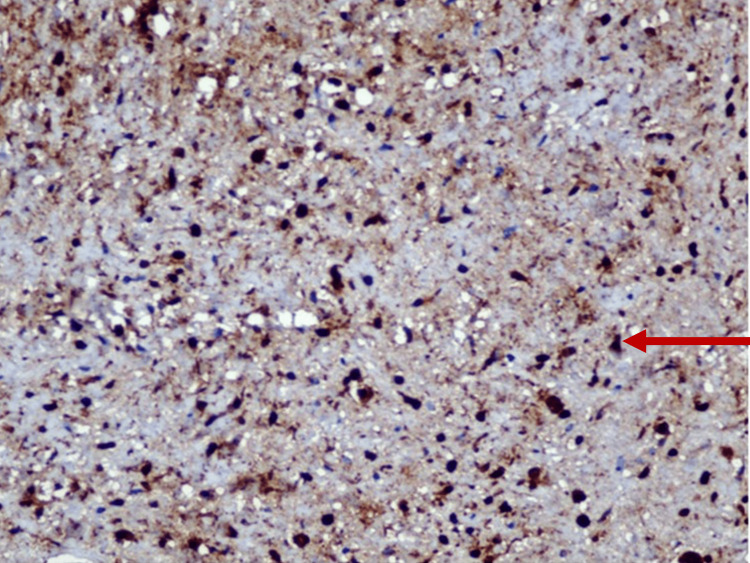
Immunohistochemistry positive for S-100 at 40×.

No dysplastic tissue or mitotic figures were seen, ruling out malignancy. Immunohistochemistry was suggested to confirm the diagnosis. Staining of the sample was found positive for S-100 protein, which was highly suggestive of neurofibroma (Figure 6).

**Figure 7:**
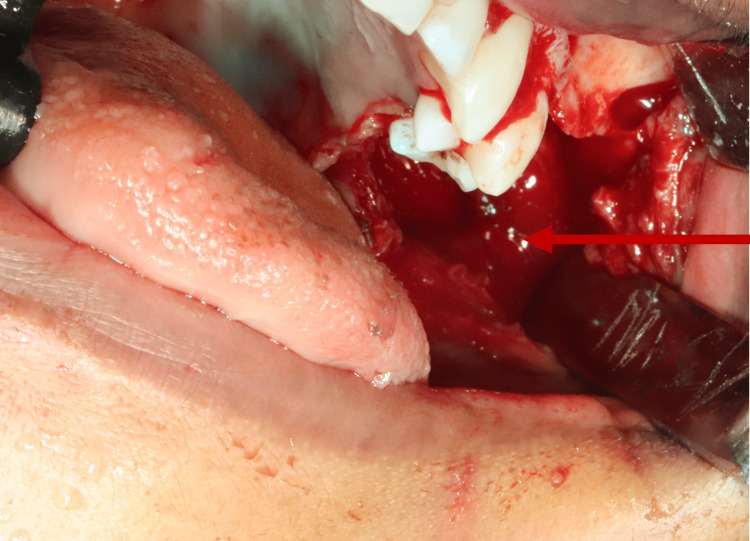
Intraoperative image showing tumor removal.

**Figure 8:**
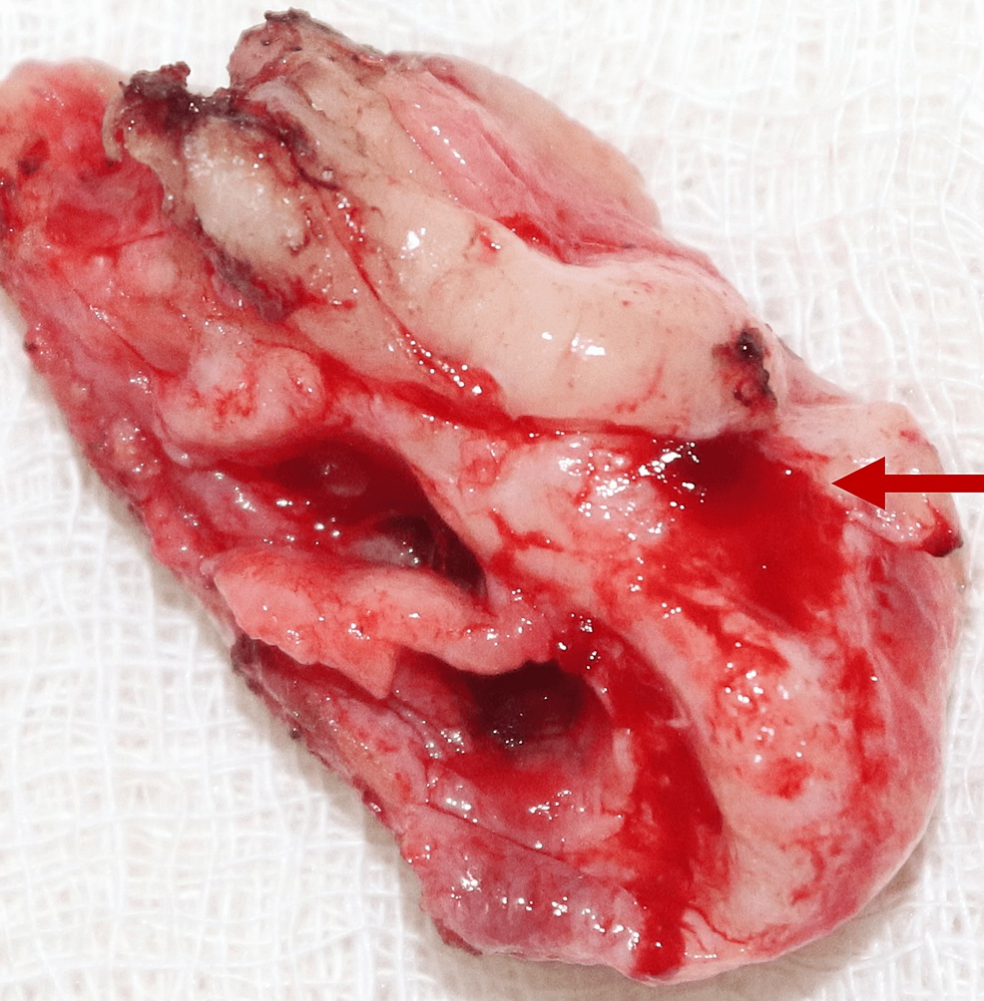
Surgical specimen showing the excised tissue.

The excision of the tumor was done under general anesthesia (Figures 7, 8).

**Figure 9:**
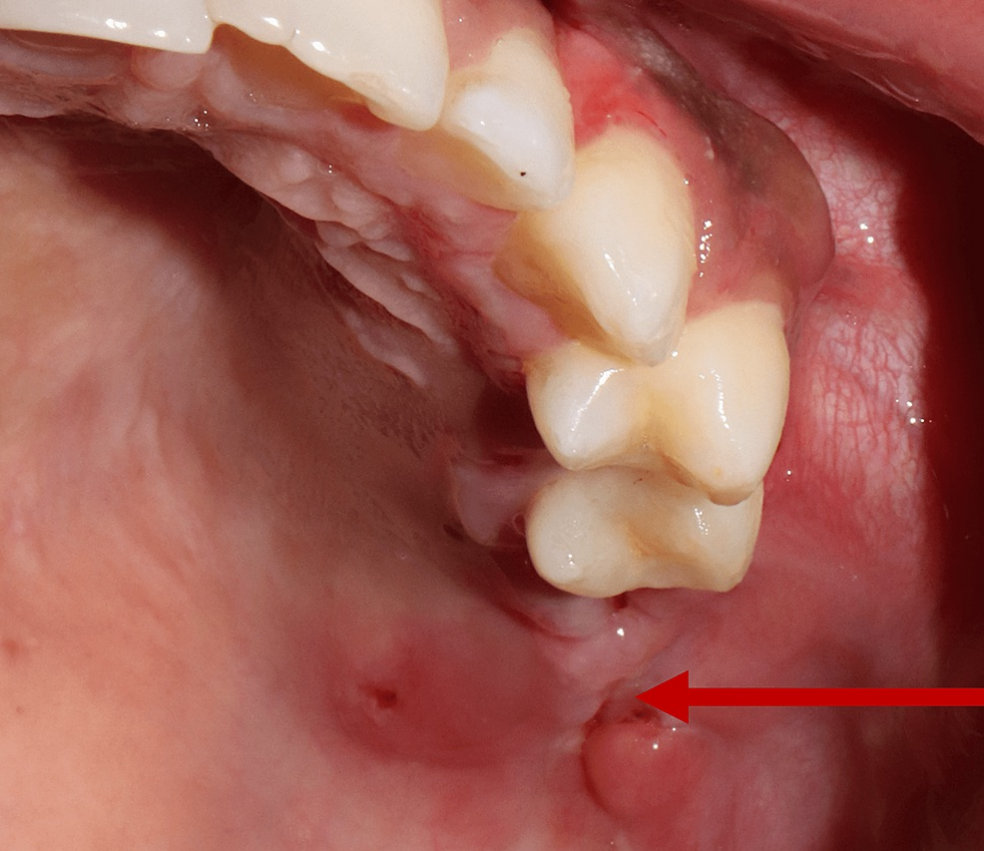
Postoperative image showing the completely healed surgical site.

The osteolytic bone was resected. The surgical site was closed with a vicryl (3-0) suture. The patient was regularly followed up at B.R. Ambedkar Institute of Dental Sciences. Three years of follow-up showed no signs of recurrence (Figure 9).

## Discussion

Neurofibromas most commonly occur in the skin. They usually occur as a local manifestation of von Recklinghausen’s disease. Neurofibromatosis is a genetically driven autosomal dominant condition. The estimated prevalence is one per 2,000 live births [2]. It is associated with multiple neurofibromas, skin pigmentation, and bone abnormalities. However, it rarely occurs as a solitary oral lesion. Solitary neurofibroma is a relatively circumscribed, slow-growing, non-encapsulated tumor. It arises from the nerve and consists of Schwann cells, perineural cells, and collagen tissues. The head and neck regions account for approximately 25% of the neurofibromas and only 6% of the tumors are reported in the oral cavity [3,4]. The reported incidence of neurofibroma in the paranasal sinus is only 4%, and in the maxillary sinus, it is even rarer.

Most neurofibromas of the ethmoid and maxillary sinus arise from the ophthalmic or maxillary branch of the trigeminal nerve [5]. In our case, as the tumor was present in the maxillary molar region, it might have originated from the posterior superior alveolar nerve.

The pathogenesis of solitary neurofibroma is unknown. However, it is assumed that solitary neurofibroma is a hyperplastic hamartomata malformation rather than neoplastic in origin [6]. Neurofibromas show strong positivity for S-100 protein on immunohistochemistry.

Neurofibromas usually do not cause bone destruction but are usually seen at an advanced stage. Therefore, a CT scan is useful to see bone destruction [7,8]. However, in our case, bone resorption was seen at only six months of presentation. MRI is useful in diagnosing neurofibroma as it shows a hyperintense signal in T2-weighted images and an isointense to hypointense signal in T1-weighted images. Heterogenous contrast enhancement is typically seen in neurofibroma [9]. MRI findings in our case also showed heterogenous enhancement consistent with neurofibroma.

The differential diagnoses of neurofibroma include schwannoma, traumatic neuroma, benign fibrous histiocytoma, desmoplastic melanoma, and malignant schwannoma. Verocay bodies are characteristic of schwannoma, while mast cell presence and a fine collagen matrix are characteristic of neurofibroma [10]. Traumatic neuromas have a positive traumatic history. Desmoplastic melanomas have junctional melanotic proliferation.

The standard treatment for benign nerve sheath tumors is enucleation. However, the treatment of solitary neurofibroma is excision as the tumor is not encapsulated and might infiltrate into the surrounding tissue.

The malignant potential of solitary neurofibroma is negligible, unlike that of neurofibromatosis. Neurofibromatosis can transform into neurofibrosarcoma in 5-16% of cases. Therefore, a long follow-up is necessary.

## Conclusions

Solitary neurofibroma of the maxillary sinus is a very rare presentation. Neurofibroma in the maxilla with early bone destruction is reported for the first time in the literature. The localization is exceptional and confusing with other neoplasms of the maxillary sinus. Heterogenous enhancement in the MRI helped guide the diagnosis. The histopathological findings and immunohistochemical analysis finally led to a confirmed diagnosis.
